# Retinal Detachment Complicating Endogenous Endophthalmitis in a COVID-19 Patient With Superimposed *Streptococcus pneumoniae* Bacteremia

**DOI:** 10.1155/crdi/8875730

**Published:** 2025-07-12

**Authors:** Madiha Hijazi, Jana Kotaich, Alaa Fares, Safaa Ghanem, Ghinwa Dakdouki

**Affiliations:** ^1^Department of Diagnostic Radiology, American University of Beirut, Beirut 1107, Lebanon; ^2^Faculty of Medical Sciences, Lebanese University, Hadath, Lebanon; ^3^MEDICA Research Investigation, Hadath, Lebanon; ^4^Department of Internal Medicine, Hammoud Hospital University Medical Center, Saida, Lebanon; ^5^Faculty of Medicine, Beirut Arab University, Beirut, Lebanon; ^6^Department of Internal Medicine, Infectious Diseases Division, Hammoud Hospital University Medical Center, Saida, Lebanon

**Keywords:** bacteremia, COVID-19, endogenous endophthalmitis, retinal detachment, *Streptococcus pneumoniae*

## Abstract

Endophthalmitis is a rare, vision-threatening ocular infection. During the COVID-19 pandemic, the widespread use of immunosuppressive agents—particularly corticosteroids—and prolonged hospital stays have been associated with an increased risk of secondary bacterial infections, including ocular involvement. One such opportunistic pathogen is *Streptococcus pneumoniae*. Among the rare but severe complications of endogenous endophthalmitis is retinal detachment (RD), which often results in a poor visual prognosis. We present a unique case of RD secondary to endogenous endophthalmitis in a COVID-19 patient with *S. pneumoniae* bacteremia. This case highlights the importance of early ophthalmologic evaluation in patients with ocular symptoms during or after COVID-19 infection to ensure timely intervention and improve clinical outcomes.

## 1. Introduction

Coronavirus disease 2019 (COVID-19), caused by the SARS-CoV-2 virus, has been associated with a wide spectrum of systemic and organ-specific manifestations [1]. Since its emergence in late 2019, over 750 million confirmed cases and more than 6.9 million deaths have been reported worldwide as of early 2024 [1]. In addition to respiratory symptoms, COVID-19 has shown neurological, cardiovascular, gastrointestinal, and ocular involvement [2].

Ocular manifestations of COVID-19 have ranged from mild conjunctivitis to more severe conditions, including anterior uveitis, retinal vascular occlusions, and optic neuritis [2]. Conjunctivitis remains the most commonly reported ocular symptom, with an incidence of approximately 1%–3% in infected individuals [2]. Endophthalmitis is rarely encountered and is associated with devastating complications, mainly vision loss, especially in patients with underlying systemic infections or immune compromise [2].

Endophthalmitis is a rare ocular infection that involves the inner layers of the eye and intraocular fluids and is classified according to the route of infection into exogenous, the most common form, and endogenous, which is seldom encountered and results from hematogenous seeding of the eye by a bacterial or fungal source [3]. Although endogenous cases account for only 2%–15% of all endophthalmitis cases, they can result in profound visual loss if not promptly diagnosed and treated [3].

Presenting symptoms of endophthalmitis include eyelid swelling, eye pain and redness, and blurry vision. Ophthalmic examination may reveal hypopyon, severely reduced visual acuity, and reduced red reflex [3]. Though diagnosis can be made clinically, different ocular sampling methods and imaging modalities can be used to document the diagnosis and assess associated findings [3]. Risk factors include recent systemic infections, intravenous drug use, immunosuppression, and hospitalization, particularly in intensive care settings [4].

Retinal detachment (RD) is another severe ocular complication that can occur following endophthalmitis, with an incidence of 8.3%–25% in such patients [5, 6]. RD may present acutely or in a delayed fashion (up to several months postinfection) and is associated with poor visual outcomes, with a significant proportion of patients ultimately requiring enucleation or evisceration [5, 7].

In this paper, we report the case of a previously healthy young patient presenting with unilateral endogenous endophthalmitis (EE) which was complicated by RD in the setting of *Streptococcus pneumoniae* bacteremia secondary to a COVID-19 infection.

## 2. Case Presentation

A 33-year-old previously healthy female presented to the emergency department on the 17th day of her COVID-19 infection, complaining of fever, chills, blurry vision, and pain in the left eye. She also reported associated arthralgia and myalgia. Vital signs were within normal limits except for a fever of 38.4°C. Physical examination revealed minimal conjunctival erythema and eyelid edema in the left eye, with a completely normal right eye.

Initial blood analysis revealed a white blood cell count (WBC) of 6.91 × 10^3^/μL (reference range: 4–11 × 10^3^/μL) and a markedly elevated C-reactive protein (CRP) level of 251 mg/L (normal: < 5 mg/L). Blood cultures grew *S. pneumoniae*, which was sensitive to vancomycin, levofloxacin, and penicillin (MIC < 0.06 μg/mL).

The patient was admitted to the COVID-19 ward and initiated on intravenous levofloxacin (Tavanic 500 mg, Sanofi Aventis, Germany), intravenous methylprednisolone sodium succinate (Solu-Medrol 40 mg, Pfizer, Belgium), and ofloxacin 3 mg/mL eye drops (Oflox, Allergan, Ireland), targeting suspected conjunctivitis and pneumonia.

**Figure 1:**
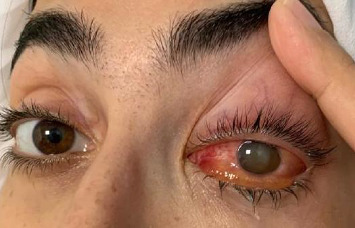
Unilateral endogenous endophthalmitis (EE) of the left eye. External examination of the right eye reveals conjunctival injection, marked ciliary flush, and diffuse scleral hyperemia. The anterior chamber appears hazy with a hypopyon partially obscuring the lower pupillary margin. Corneal edema and loss of the normal red reflex are also noted, suggesting significant intraocular inflammation. These findings are consistent with acute endophthalmitis.

Despite initial clinical and hemodynamic improvement, the patient developed worsening ocular symptoms 3 days later, including a temperature spike to 39.5°C, photophobia, and severe left eye pain. Ophthalmologic evaluation revealed eyelid edema, diffuse conjunctival erythema, a nonreactive and visible pupil, reduced red reflex, clear discharge, and hypopyon. Visual acuity was significantly reduced to hand motion perception in the affected eye, while the right eye remained normal (Figure 1). The patient denied any recent ocular trauma or ophthalmologic procedures.

**Figure 2:**
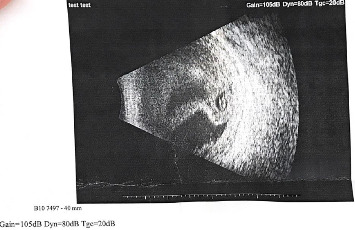
Ultrasound of the left eye: This image shows inflammatory debris present—the vitreous chamber shows dense vitreous opacities with signs of retinal detachment. Inflammatory debris likely indicating vitritis, a key feature of endophthalmitis.

Repeat labs showed elevated WBC (9.38 × 10^3^/μL) and CRP (224.4 mg/L). EE was suspected. However, the patient refused a vitreous tap for culture. Ocular ultrasonography of the left eye revealed a folded membrane within the vitreous chamber, consistent with RD (Figure 2).

Given the patient's refusal of any intraocular interventions, systemic management was continued with intravenous levofloxacin and methylprednisolone every 8 hours, along with topical ofloxacin drops. After two additional days of treatment, the patient chose to leave the hospital against medical advice.

## 3. Discussion

*S. pneumoniae* is a significant but relatively rare cause of ocular infectious diseases. Among bacterial pathogens, *S. pneumoniae* endophthalmitis carries one of the poorest prognoses, often leading to severe visual impairment despite timely treatment [8].

Studies have shown that any alteration in the blood ocular barrier promotes invasion of organisms into ocular tissue [3]. Although bacterial agents account for the majority of endophthalmitis cases, *S. pneumoniae* remains an uncommon but particularly aggressive organism, often associated with devastating visual outcomes [9, 10]. In a study conducted by Chen et al. [8] only 8% of patients with *S. pneumoniae* endophthalmitis achieved a visual acuity of 20/400 or better, and 26% required evisceration or enucleation. The organism was commonly isolated from vitreous and anterior chamber samples; however, culture positivity tends to be low, particularly when systemic antibiotics are administered prior to ocular sampling [4].

The exact mechanism by which COVID-19 leads to ocular involvement remains unclear, and consensus has not yet been reached regarding viral accumulation in ocular secretions [2]. EE cases have been reported in COVID-19 patients where an etiological agent was detected in the majority of cases [11, 12]. In a case series by Bilgic et al. [12], one patient had positive COVID-19 PCR of the vitreal fluid, however, *Klebsiella pneumoniae* was isolated from his blood culture.

Diagnosis of endophthalmitis involves imaging, ocular sampling, and systemic assessment [3]. Management generally follows a three-pronged approach: (1) elimination of the infectious source via systemic and intravitreal antibiotics, (2) control of inflammation using corticosteroids, and (3) supportive care, including cycloplegic agents and hypertonic saline in cases of corneal edema [3].

RD is a recognized complication of endophthalmitis and contributes significantly to poor visual outcomes [5]. The diagnosis typically involves evaluation of the retina using techniques such as indirect ophthalmoscopy along with ultrasonography, CT, or MRI which can be used for further assessment [7]. Treatment options for RD often involve surgical intervention to repair the retinal tear and reattach the retina to its normal position [7]. As mentioned previously by Wang et al. [5], approximately one-third of patients who develop RD secondary to endophthalmitis ultimately require evisceration or enucleation.

To our knowledge, this is the first reported case of RD following EE in a COVID-19 patient with *S. pneumoniae* bacteremia. The initial presentation included nonspecific symptoms such as unilateral eye pain and redness, which led to a preliminary diagnosis of conjunctivitis. As the clinical picture evolved and blood cultures identified *S. pneumoniae*, the diagnosis of EE was considered. Although a vitreous tap was advised to confirm intraocular infection, the patient declined the procedure. Ocular ultrasonography was therefore performed, which revealed RD.

Systemic and topical antibiotics were initiated. However, intravitreal antibiotic therapy and surgical intervention were not pursued due to the patient's refusal. To manage intraocular inflammation, intravenous methylprednisolone sodium succinate 40 mg was administered every 8 hours, in alignment with evidence-based recommendations [3].

## 4. Conclusion

COVID-19 infection, coupled with bacterial or fungal hematogenous spread, has been associated with various ocular complications, often presenting with nonspecific symptoms. This case highlights a rare but severe manifestation of EE with RD due to *S. pneumoniae* superinfection in a COVID-19 patient. RD, as a complication of EE, poses a significant risk of irreversible vision loss, emphasizing the importance of early detection and intervention.

Given the overlapping symptoms of common ocular conditions, clinicians must maintain a high index of suspicion for sight-threatening infections in post-COVID-19 patients presenting with ocular discomfort. Prompt ophthalmologic evaluation, imaging, and systemic assessment are crucial in guiding timely management.

## Data Availability

The data that support the findings of this study are available on request from the corresponding author. The data are not publicly available due to privacy or ethical restrictions.
