# Correction to “Retinal Detachment Complicating Endogenous Endophthalmitis in a COVID‐19 Patient With Superimposed *Streptococcus pneumoniae* Bacteremia”

**DOI:** 10.1155/crdi/9768189

**Published:** 2026-02-10

M. Hijazi, J. Kotaich, A. Fares, et al., “Retinal Detachment Complicating Endogenous Endophthalmitis in a COVID‐19 Patient With Superimposed *Streptococcus pneumoniae* Bacteremia,” *Case Reports in Infectious Diseases* (2025): 2025 8875730, https://doi.org/10.1155/crdi/8875730.

In the article titled, “Retinal Detachment Complicating Endogenous Endophthalmitis in a COVID‐19 Patient With Superimposed *Streptococcus pneumoniae* Bacteremia,” Hannah Chehade, Rana Farhat, Leila Al Abed were missing in the author list. The corrected author list, affiliations, and author contributions are provided below.

Madiha Hijazi,^1^ Jana Kotaich,^2,3^ Alaa Fares,^4^ Safaa Ghanem,^5^ Hannah Chehade,^5^ Rana Farhat,^5^ Leila Al Abed,^5^ Ghinwa Dakdouki^6^

^1^Department of Diagnostic Radiology, American University of Beirut, Beirut 1107, Lebanon

^2^Faculty of Medical Sciences, Lebanese University, Hadath, Lebanon

^3^MEDICA Research Investigation, Hadath, Lebanon

^4^Department of Internal Medicine, Hammoud Hospital University Medical Center, Saida, Lebanon

^5^Faculty of Medicine, Beirut Arab University, Beirut, Lebanon

^6^Department of Internal Medicine, Infectious Diseases Division, Hammoud Hospital University Medical Center, Saida, Lebanon

**Author Contributions**

Hannah Chehade: data interpretation, literature review, and manuscript drafting.

Rana Farhat: clinical data collection, patient management, and writing–original draft.

Leila Al Abed: literature review, critical revision of the manuscript for intellectual content, and writing–original draft.

We apologize for these errors.

